# Establishing the role of BRCA1 in the diagnosis, prognosis and immune infiltrates of breast invasive cancer by bioinformatics analysis and experimental validation

**DOI:** 10.18632/aging.205366

**Published:** 2024-01-13

**Authors:** Leilei Li, Shuangyan Li, Xuyang Zhang, Liying Mei, Xueqin Fu, Min Dai, Na Wei

**Affiliations:** 1 Department of Pathology, Kunming Medical University, Kunming 650500, Yunnan, China; 2 Department of Oncology, Kunming Medical University, Kunming 650500, Yunnan, China; 3 Department of Hepatobiliary, Second Affiliated Hospital of Zunyi Medical University, Zunyi 563000, Guizhou, China; 4 Department of Breast Surgery, Guizhou Provincial People’s Hospital, Guiyang 550002, Guizhou, China

**Keywords:** breast invasive cancer, breast cancer susceptibility gene 1, prognosis, immune cells, methylation

## Abstract

Background: Breast cancer susceptibility gene 1 (BRCA1) is a well-known gene that acts a vital role in suppressing the growth of tumors. Previous studies have primarily focused on the genetic mutations of BRCA1 and its association with hereditary breast invasive carcinoma (BRCA). However, little research has been done to investigate the relationship between BRCA1 and immune infiltrates and prognosis in BRCA.

Methods: We obtained the expression profiles and clinical information of patients with BRCA from the Cancer Genome Atlas (TCGA) database. The levels of the BRCA1 gene between BRCA tissues and normal breast tissues were compared through the Wilcoxon rank-sum test. Additionally, we performed WB and RT-qPCR techniques to detect the expression of BRCA1. We conducted functional enrichment analyses. Furthermore, we assessed immune cell infiltration using a single-sample gene set enrichment analysis. The methylation status of the BRCA1 gene was analyzed using the UALCAN and MethSurv databases. The Cox regression analysis and (KM) Kaplan-Meier method were employed to determine the prognostic value of BRCA1. In order to provide a practical tool for predicting the overall survival rates at different time points, we also constructed a nomogram.

Results: Our analysis revealed that the expression of BRCA1 was significantly higher in BRCA tissues compared to normal tissues. Furthermore, this increased level of BRCA1 was found to be associated with specific BRCA subtypes, including T2, stage II, ER positive, ect. Importantly, the overexpression of BRCA1 was shown to be a negative prognostic marker for the overall survival rates of BRCA patients. Moreover, low methylation status of the BRCA1 gene was related to a poorer prognosis. Furthermore, our results indicated that high levels of BRCA1 are related to a decrease in level of killer immune cells, such as natural killer (NK) cells, macrophages, CD8^+^ T cells, and plasma-like dendritic cells (pDCs) within the tumor microenvironment.

Conclusions: Our study is the first to provide evidence indicating that the presence of BRCA1 can serve as a reliable marker for both diagnosing and determining the prognosis of BRCA. Moreover, BRCA1 acts as a crucial indicator of the cancer’s potential to infiltrate and invade the immune system, which has important implications for developing targeted therapies in BRCA.

## INTRODUCTION

Breast invasive cancer (BRCA) is the most common malignant tumor in women across the world. It accounts for approximately 11.7% of all cancer cases, or around 2.3 million new cases in the year 2020 [1, 2], making it a major public health concern. Over the years, BRCA has garnered significant attention from researchers and medical professionals, leading to extensive exploration of its causes, progression, and potential treatment options. In recent decades, remarkable progress has been made in diagnosing and treating BRCA [3]. Several sensitive and efficient approaches have been developed, reducing the mortality rates associated with BRCA. However, despite these improvements, BRCA exhibits inherent heterogeneity, meaning that each patient’s tumor can have distinct characteristics and respond differently to treatment. Unfortunately, despite the advancements in diagnosis and treatment, the prognosis for most BRCA patients still remains poor. This highlights the urgent need for the development and implementation of more effective diagnostic and therapeutic methods.

Breast cancer susceptibility gene 1 (BRCA1) is a widely recognized gene that is responsible for suppressing tumor growth [4–6]. It is commonly found to be mutated in individuals with a family history of breast and ovarian cancers [7]. Studies have shown that mutations in BRCA1 are detected in approximately 20% to 25% of hereditary BRCA cases and 5% to 10% of all BRCA cases [8]. Furthermore, it has been observed that a loss of heterozygosity (LOH) of BRCA1 is frequently observed in high-grade BRCA [9, 10]. BRCA1 plays a crucial role in maintaining genomic stability and is involved in various cellular pathways [6]. These pathways include DNA damage repair, activation of cell-cycle checkpoints in response to DNA damage, regulation of gene transcription, protein ubiquitination, chromatin remodeling, and apoptosis [11]. Despite extensive research on BRCA1, its association with prognosis and immune infiltrates in BRCA has not been reported yet.

The objective of this study is to utilize interactive tools to explore the transcriptional expression and prognostic significance of BRCA1 in BRCA. By employing bioinformatics techniques, we aim to investigate the relationship between BRCA1 expression and its clinical pathological features, prognostic implications, and immune cell infiltration in BRCA. Such investigations have the potential to aid clinicians in improving the treatment strategies and overall prognosis for patients diagnosed with BRCA.

## MATERIALS AND METHODS

### Collection of samples

From 2013 to 2023, a total of thirty BRCA tissues were collected from individuals who were undergoing breast cancer resection at Breast Surgery Department of Guizhou Provincial People’s Hospital. The clinical information of BRCA patients is displayed in Table 1. The selection criteria for inclusion in the work required a confirmed diagnosis of BRCA through pathology examination. The entire process of sample collection was conducted in accordance with the ethical guidelines set forth by the ethics committee of the Guizhou Provincial People’s Hospital, ensuring the protection of the patients’ rights and well-being.

**Table 1:** Clinical features of patients.

**Features**	**Variables**	**No. (%)**
Age	< 60	10 (33)
	≥ 60	20 (67)
Gender	Female	30 (100)
Molecular subtype	Luminal A	5(17)
	Luminal B	5(17)	HER-2(+)	10(33)	TNBC	10(33)
	Lymphatic metastasis	Yes	18(60)
		No	12(40)
Distant metastasis	Yes	8(27)
	No	22 (73)

### Real-time fluorescent quantitative PCR assay (RT-qPCR)

Total RNA was isolated from tissues or cells according to the instructions of the RNA extraction kit (LS1040, Promega, Shanghai, China). Next, a fixed one-step RT-PCR kit (A6120, Promega, Shanghai, China) was utilized to synthesize cDNA. RT-qPCR assay was employed utilizing the SYBR Green SuperMix system (TSE201, Tsingke Bio Technology, Beijing, China). The changes in gene levels were estimated by the 2^-ΔΔCT^ method. The primer sequences utilized for assay were displayed in Table 2.

**Table 2:** The primer sequences for qPCR.

**Primers for validated genes**	**Prime sequence (5’-3’)**	
**Gene**	**Forward**	**Reverse**
GAPDH	TATGACAACAGCCTCAAGAT	AGTCCTTCCACGATACCA
BRCA1	CAGAGGACAATGGCTTCCATG	CTACACTGTCCAACACCCACTCTC

### Protein isolation and Western blotting

The total protein was isolated from cells using the protein extraction kit (R0010, Solarbio, Beijing, China). To determine the protein concentration, the BCA protein assay kit (P0010, Beyotime, Shanghai, China) was employed. For the experiment, primary antibodies specifically targeting β-actin and BRCA1 were from ZSGB-BIO, China (TA-09, 1:1000) and HUABIO, China (HA500015, 1:1000), respectively. These primary antibodies were then incubated at 4° C overnight. After the primary antibodies were incubated with the samples, the next step involved diluting the secondary antibodies. Specifically, either goat anti-mouse IgG-HRP or goat anti-rabbit IgG-HRP antibodies (ZSGB-BIO, Zhongshan, China) were mixed in a dilution ratio of 1:10000 (37° C for 1 hour).

### Data collection from TCGA database

All mRNA expression data and clinical data of BRCA patients included in this study were sourced from two major databases, namely the TCGA (The Cancer Genome Atlas) database and the GTEx (Genotype-Tissue Expression) database. The TCGA database is a collaborative effort by multiple institutions and contains comprehensive molecular data from tumor samples, while the GTEx database provides genomic and expression data from normal tissues. By utilizing these databases, this study ensured a robust and representative dataset that encompasses both cancerous tissue samples and normal tissue samples, allowing for a comprehensive analysis of the mRNA expression profiles in BRCA patients and their correlation with clinical outcomes.

### Cell culture

The BRCA cell lines of human, including MCF-7, T47D, MDA-MB-231, and HCC-1806, were obtained from The Center for Molecular and Cellular Sciences in Shanghai, China. These cell lines were cultured in RPMI-DMEM which were purchased from VivaCell in Shanghai. Additionally, the culture media were supplemented with 1% penicillin-streptomycin solution from Beyotime in Shanghai and 10% fetal bovine serum from VivaCell. On the other hand. All the cells mentioned above were cultured in a controlled environment at 37° C with an atmosphere containing 5% CO_2_.

### Differential expression analysis of BRCA1

Based on the minimum P-value of BRCA1 expression, the researchers categorized the cancer TCGA patients into two groups: BRCA1 high expression group and BRCA1 low expression group. To further investigate the differences in gene expression between these two groups, the researchers utilized the R package DESeq2 developed by Love et al. in 2014 [12]. They set the threshold for differentially expressed genes (DEGs) as adjusted p-value<0.05 and | log_2_-fold change (FC) |>1. Additionally, the researchers aimed to evaluate the correlation between the expression of the top 10 DEGs and BRCA1. To do this, they employed Spearman correlation analysis, a statistical method used to measure the strength and direction of the monotonic relationship between two variables.

### Enrichment analysis

GSEA, which stands for Gene Set Enrichment Analysis, is a computational method used to standardize RNA Seq data obtained from TCGA. This analysis tool, available at the website MSigDB, allows researchers to investigate the biological functions of BRCA1. To identify potential biological functions, we utilized GO (Gene Ontology) terminology and KEGG (Kyoto Encyclopedia of Genes and Genomes) pathway enrichment analysis. These analyses are performed using a gene clustering analyzer implemented in the R programming language. The GO terminology is categorized into three aspects: biological process (BP), molecular function (MF), and cellular composition (CC). By analyzing the genes associated with BRCA1, researchers can gain insights into the specific biological processes, molecular functions, and cellular components related to this gene. In addition, KEGG pathway enrichment analysis allows researchers to explore the potential pathways in which BRCA1 is involved. The KEGG pathway database catalogs various biological pathways and provides a comprehensive understanding of the molecular interactions and signaling events related to specific genes or gene sets. To ensure the reliability of the enrichment results, two conditions must be met: an error detection rate (FDR) of less than 0.05 and a nominal p-value of less than 0.05. These thresholds help researchers identify statistically significant enrichment results, indicating that the observed biological functions and pathways associated with BRCA1 are unlikely to occur by chance.

### Analysis of immune cells infiltration

To determine the immune infiltration level, 24 immune cells were utilized in the analysis. The relative enrichment fraction of these immune cells in BRCA was measured using a single sample Gene Set Enrichment Analysis (GSEA) approach, which was conducted utilizing the R package GSVA [13]. Additionally, the relation between BRCA1 level and 24 immune cells was discussed utilizing Spearman correlation analysis. Furthermore, the differences in immune infiltration levels between BRCA1 high- and low- groups were evaluated using the Wilcoxon rank sum test.

### DNA methylation analysis

To discuss the potential mechanism of BRCA1 in BRCA development, we utilized the UALCAN database [14], which provides a comprehensive analysis of cancer transcriptome data. Specifically, we focused on analyzing the methylation status of the BRCA1 promoter, a region responsible for regulating the expression of the BRCA1 gene. Furthermore, to assess the potential clinical significance of BRCA1 methylation levels, we employed the MethSurv database [15] — an invaluable online resource for conducting multivariate survival analysis based on DNA methylation profiles. By integrating clinical outcome data with methylation information, we aimed to determine whether the methylation status of BRCA1 could serve as a prognostic marker in cancer patients. Through these analyses, we aimed to gain insights into the role of BRCA1 methylation in cancer development, potentially providing a novel avenue for targeted therapies and personalized treatment strategies.

### Validation of the nomogram

In order to predict the overall survival probability, we conducted a multivariate Cox analysis and established a column chart based on independent prognostic factors. This column chart served as a visual representation of the relationship between these factors and the survival probability. We then used a calibration chart to assess the performance of the column chart. By comparing the observed and predicted survival probabilities, we were able to evaluate the accuracy of our predictions. Additionally, we used a consistency index (C-index) to measure the discrepancies between the predicted and observed survival probabilities. This allowed us to quantify the effectiveness of our column chart in predicting overall survival. To create the column charts and calibration charts, we utilized the R package called RMS. With the help of RMS, we were able to visualize the relationship between the independent prognostic factors and the overall survival probability, allowing for a better understanding of the impact of these factors on survival outcomes. To further evaluate the accuracy of our prediction, we employed the timeROC software package. This software enabled us to conduct time-dependent receiver operating characteristic (ROC) curves, which provided a comprehensive assessment of the predictive performance over time. By analyzing the ROC curves, we were able to determine the accuracy of our prediction at different time points and assess if our model was capable of accurately predicting survival probabilities at different stages of the disease. This helped us assess the reliability and applicability of our predictions in a real-world clinical setting.

### Survival analysis

In order to analyze the survival data, we employed the Kaplan Meier method and logarithmic rank test. The cutoff value for this analysis was set at the minimum P-value of BRCA1 expression, a gene of interest. To assess the impact of various clinical variables on patient prognosis, both univariate and multivariate Cox regression analysis were performed. In the univariate Cox regression analysis, we identified a prognostic variable with a significance level of p<0.1. This significant variable was further considered in the multivariate Cox analysis. To present the results in a visually appealing manner, we utilized the R software package ggplot2 to generate forest plots, which provided a graphical representation of the multivariate Cox regression results.

### Statistical analysis

SPSS 22.0 software was adopted for data analysis. The expression difference between BRCA and normal tissues were analysed with the Wilcoxon signed-rank test. One-way analysis of variance was employed to perform comparisons between two groups. P <0.05 was deemed to be statistically significant.

### Data availability statement

The data that support the findings of this study are available on request from the corresponding author.

### Consent for publication

All authors have read and agreed to the published version of the manuscript.

## RESULTS

### High expression of BRCA1 in BRCA

**Figure 1:**
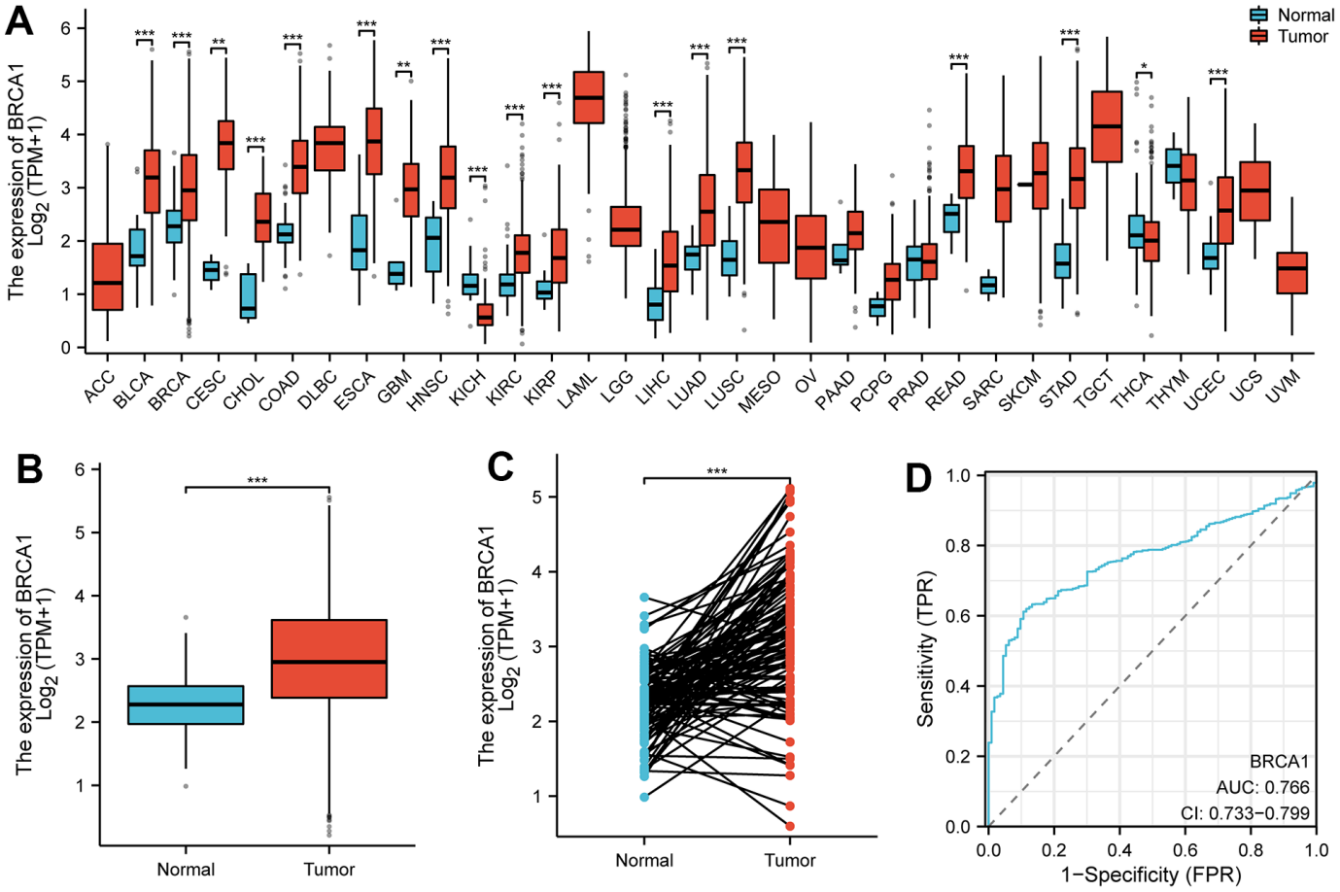
**The expression level of BRCA1 in tumors.** (**A**) BRCA1 was highly expressed in many solid tumors, including BRCA (**B**, **C**). The ROC curve area was 0.766 (**D**), indicating BRCA1 was a biomarker of diagnostic of BRCA. P-values were calculated with two-tailed unpaired Student’s t-test, *p < 0.05, **p < 0.01, ***p < 0.001.

The pan-cancer analysis showed that the level of BRCA1 was higher in most tumors compared to normal tissues, such as lung adenocarcinoma, bladder urothelial carcinoma, bile duct cancer, etc. (Figure 1A). The level of BRCA1 in BRCA was vitally higher than in normal tissue (Figure 1B). In addition, its expression was significantly higher than in paired adjacent tissues in 110 cases of BRCA tissues (Figure 1C). Furthermore, the Receiver Operating Characteristic (ROC) curve analysis indicated that the expression of BRCA1 gene in BRCA patients demonstrated a high level of accuracy in predicting the presence of the disease. The Area Under the Curve (AUC) value of the ROC curve, which measures the discriminatory power of the BRCA1 expression in distinguishing between BRCA patients and healthy individuals, was determined to be 0.766 (Figure 1D). This suggested that BRCA1 could serve as a reliable biomarker for the diagnosis of BRCA, potentially aiding in the early detection and effective management of the disease.

**Figure 2:**
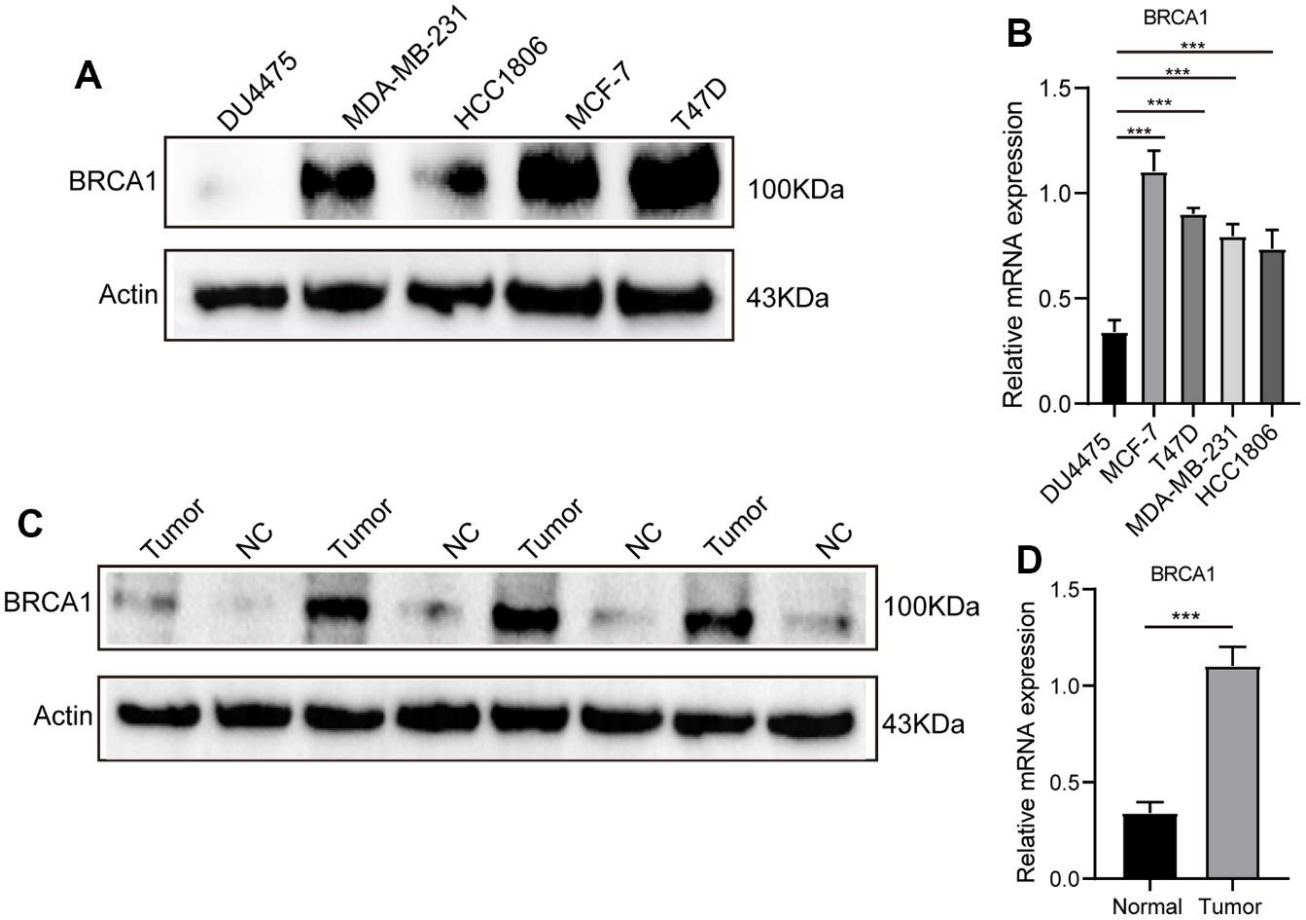
**WB and RT-qPCR were used to detect the level of BRCA1 in BRCA.** (**A**) WB and RT-qPCR (**B**) results showed that the expression of BRCA1 in breast cancer cells was higher than that in normal breast epithelial cells. (**C**) WB and RT-qPCR (**D**) results revealed that the level of BRCA1 in breast cancer tissues was higher than that in paracancerous tissues. The experiments were repeated 3 times. Data are shown as means ± SD. P-values were calculated with two-tailed unpaired Student’s t-test, *p < 0.05, **p < 0.01, ***p < 0.001.

We conducted a comprehensive analysis using various experimental techniques to further evaluate the level of BRCA1 in BRCA tissues and ascertain its significance. First, we selected a number of breast cancer cell lines and compared them to normal breast epithelial cells using WB and RT-qPCR techniques. The results from both WB and RT-qPCR clearly demonstrated that the expression of BRCA1 in breast cancer cell lines was significantly upregulated when compared to normal breast epithelial cells (Figure 2A, 2B). To further substantiate these findings, we expanded our analysis to include actual breast cancer tissues obtained from patients. We randomly selected 4 breast cancer tissues and their adjacent normal tissues for WB and RT-qPCR detection. Consistent with our previous observations, the WB and RT-qPCR results confirmed a significant increase in the expression of BRCA1 in breast cancer tissues compared to their adjacent counterparts (Figure 2C, 2D).

### Associations between BRCA1 expression and clinicopathologic variables

**Figure 3:**
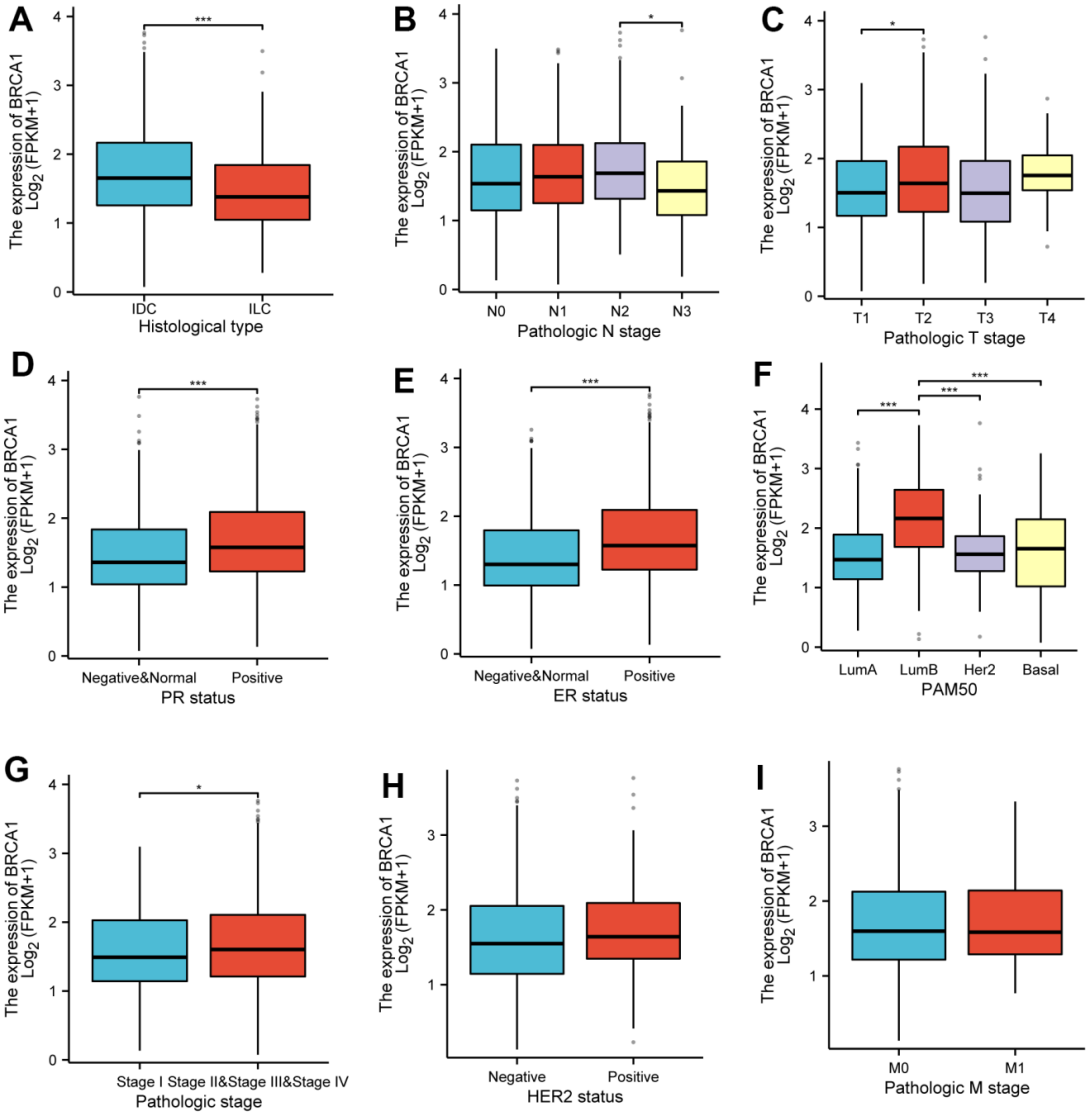
**Associations between BRCA1 expression and clinicopathological characteristics.** Data were shown for (**A**) histological type, (**B**) N stage, (**C**) T stage, (**D**) PR status, (**E**) ER status, (**F**) PAM50, (**G**) pathological stage, (**H**) HER2 status, (**I**) M stage. P-values were calculated with two-tailed unpaired Student’s t-test, *p < 0.05, **p < 0.01, ***p < 0.001.

The increased expression levels of BRCA1 in invasive ductal carcinoma (IDC) compared to invasive lobular carcinoma (ILC) (Figure 3A). Furthermore, higher BRCA1 expression was found to be significantly associated with more advanced pathological staging, specifically in the N2 compared to the N3 (Figure 3B), and in the T2 compared to the T1 (Figure 3C). The increased expression levels of BRCA1 and PR or ER positive (Figure 3D, 3E). Additionally, the analysis of the PAM50 molecular subtypes revealed that elevated BRCA1 expression was linked to specific subtypes: Luminal B subtype compared to Luminal A, Luminal B compared to human epidermal growth factor receptor 2 (HER2) subtype, and Luminal B compared to Basil subtype (Figure 3F). However, pathological stage, HER-2 status, and M stage were observed to have no significant relationship with BRCA1 expression (Figure 3G–3I).

### Prognostic value of BRCA1 in BRCA

**Figure 4:**
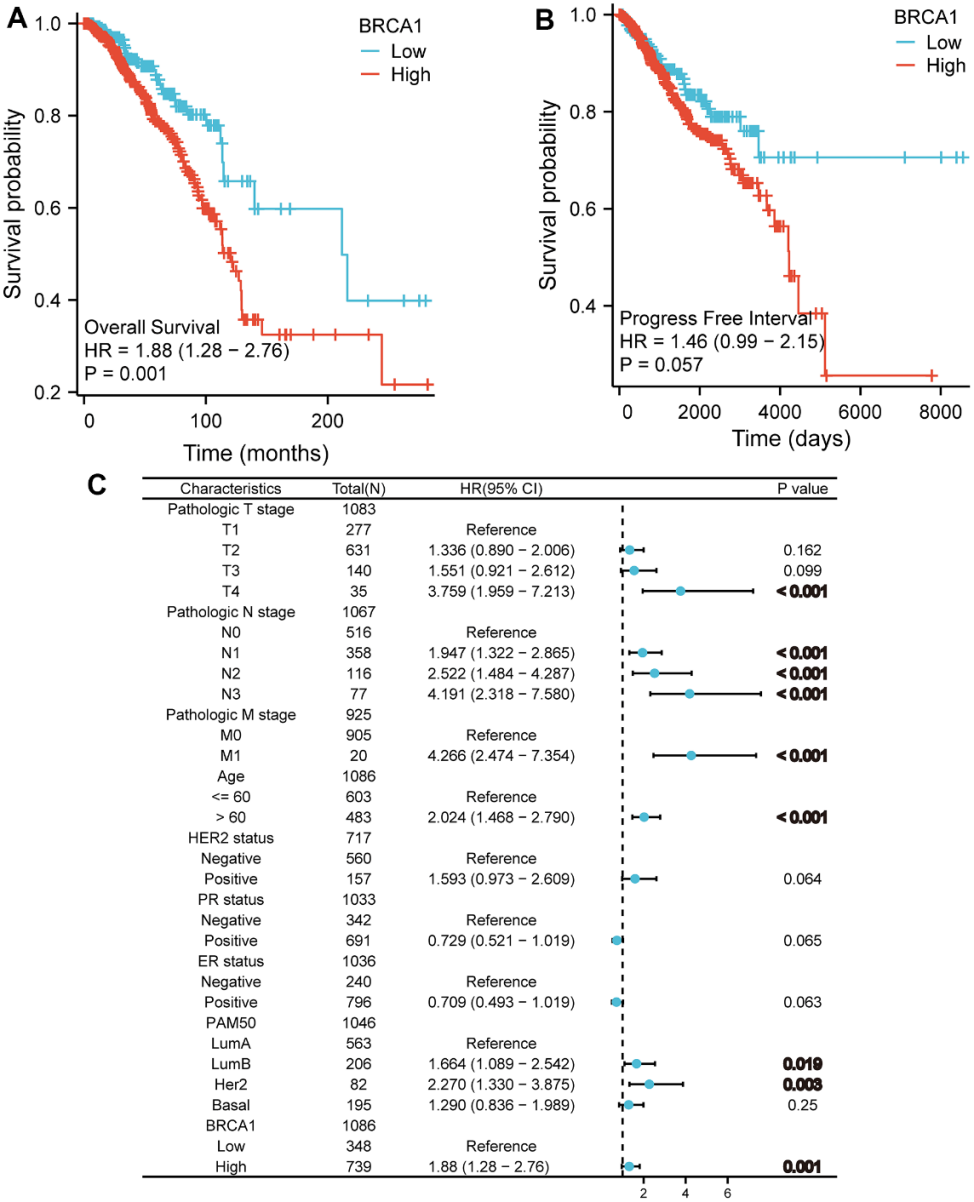
**The impact of BRCA1 level on prognosis in BRCA patients was evaluated utilizing Kaplan Meier.** (**A**) OS and (**B**) PFI for BRCA patients with high- vs low- BRCA1. (**C**) Forest map of OS with BRCA patients based on multivariate Cox analysis.

**Figure 5:**
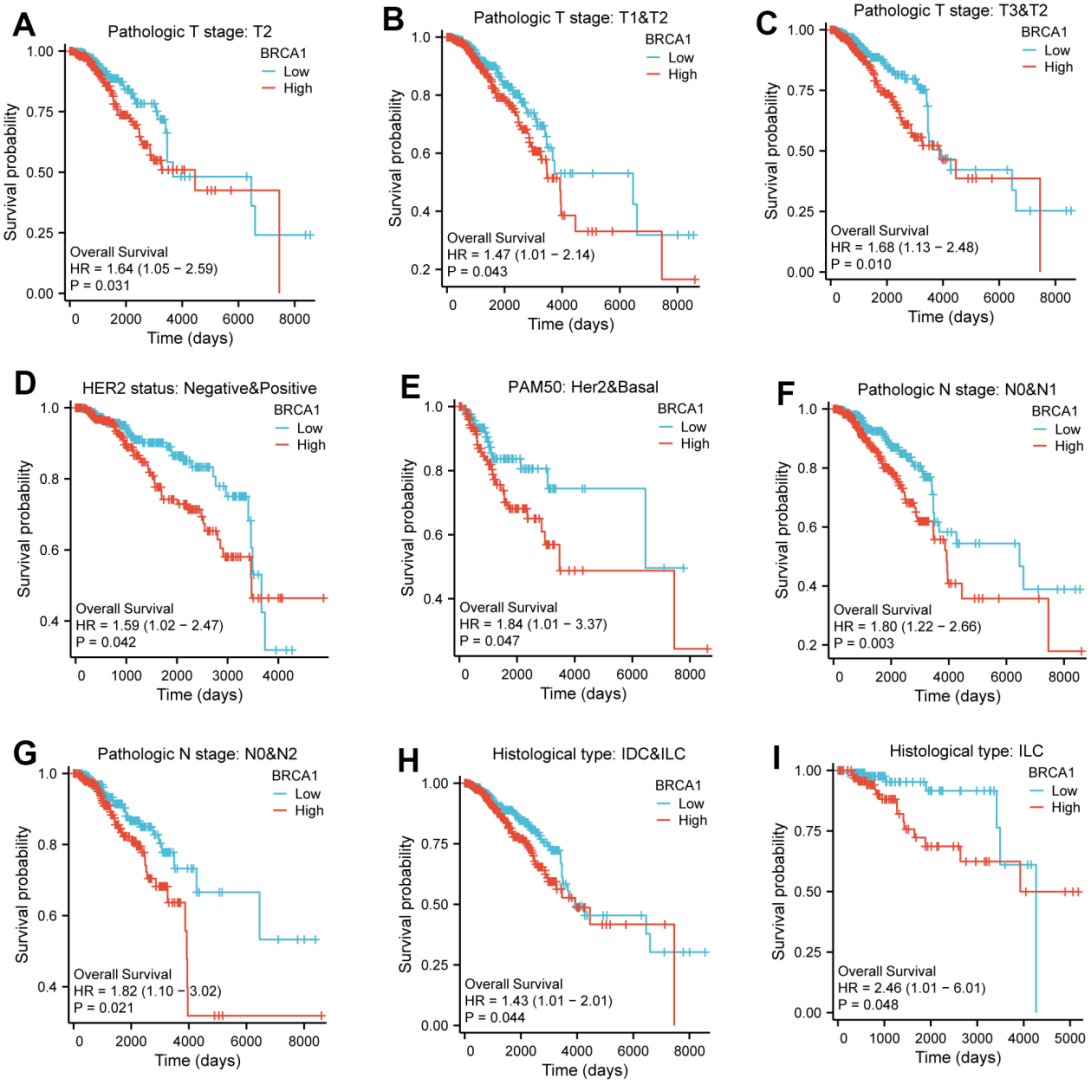
**The impact of BRCA1 level on different subgroups prognosis of patients with BRCA discussed by the Kaplan-Meier.** (**A**–**I**) OS survival curves of HER2 status, HER2 and Basel, N0 and N1, N0 and N2, IDC and ILC, ILC, T2, T1 and T2, T3 and T2 between high- and low- BRCA1 patients with BRCA.

The (KM) Kaplan Meier method was utilized to assess the association between the level of BRCA1 and the prognosis of BRCA. To classify patients based on BRCA1 expression, the minimum P-value of BRCA1 expression was used as the cutoff point. Consequently, patients were categorized into two groups: the BRCA1 high- and low- level group. Comparing the outcomes, it was observed that patients in the high expression group of BRCA1 had a worse overall survival (OS) prognosis compared to patients in the low expression group (Figure 4A). The progression free interval (PFI) of the BRCA1 high expression group also seemed to be lower (Figure 4B). Furthermore, the researchers also examined the prognosis of patients with high BRCA1 expression across various subgroups. They discovered that patients with high BRCA1 expression had significantly unfavorable prognoses in several subgroups, including those with Luminal A and Luminal B subtypes, HER2 and Basil subtypes. Additionally, patients with high BRCA1 expression had worse outcomes in subgroups with different tumor sizes (specifically T1 and T2, T3 and T2), absence and presence of lymph node metastasis (N0 and N1), age older than 60 years, patients with invasive ductal carcinoma (IDC) and invasive lobular carcinoma (ILC) (Figure 5). To obtain a more comprehensive understanding of prognostic indicators in BRCA patients, researchers employed both univariate and multivariate Cox regression analysis. Additionally, certain clinical characteristics were identified as independent factors influencing OS. The results showed that BRCA1 expression, N1 stage, N2 stage, N3 stage, M1, T4 stage, age and Luminal B and HER2 classification were independent factors of OS in BRCA patients (Figure 4C).

### Functional enrichment analysis of differentially expressed genes (DEGs) related to BRCA1

**Figure 6:**
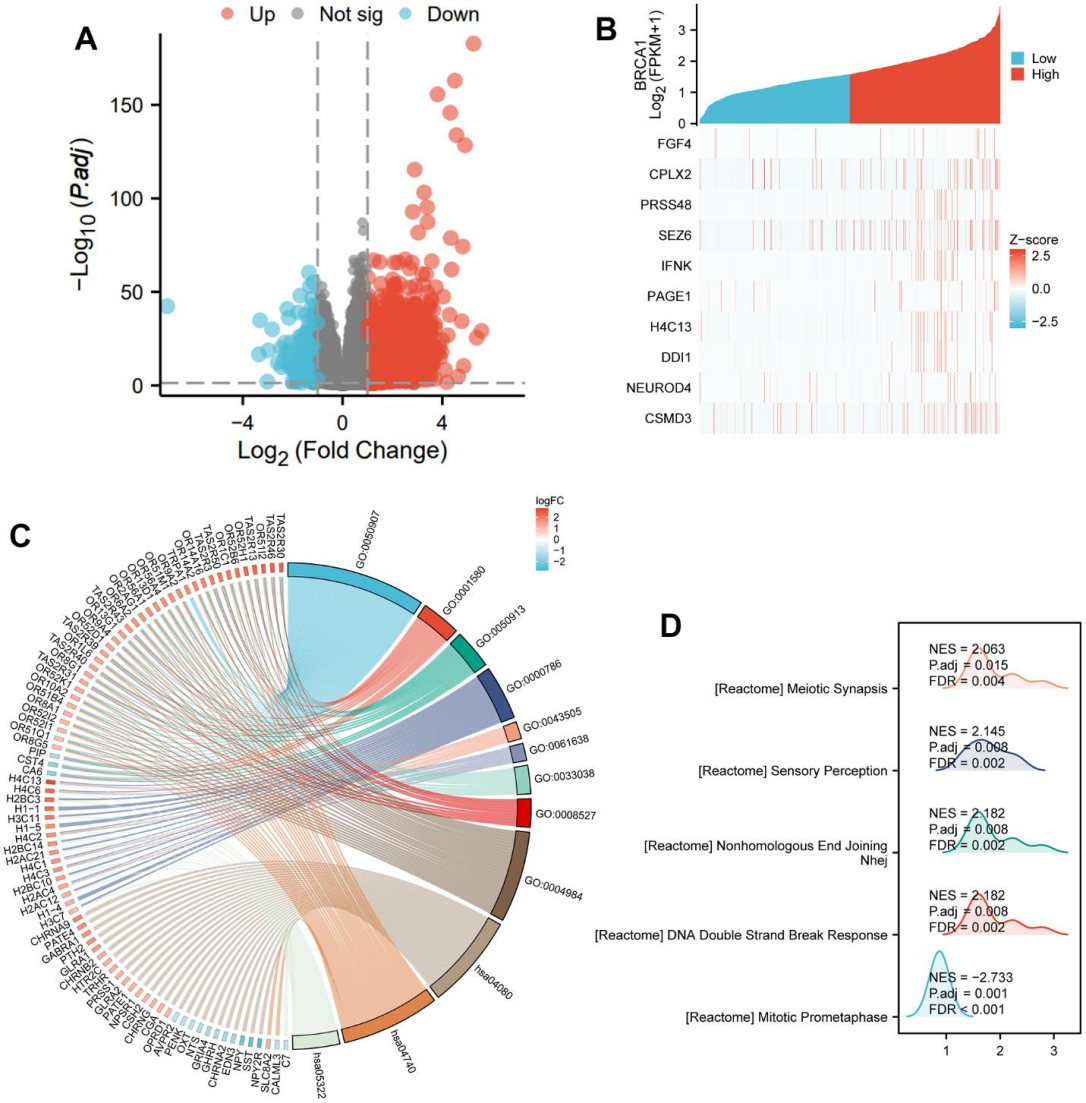
**DEGs related to BRCA1 and its functional enrichment analysis utilizing GSEA, GO and KEGG.** (**A**) Blue and red dots indicated the vitally down- and up-regulated DEGs in the Volcano plot, respectively. (**B**) The top ten DEGs positively correlated with BRCA1 level. (**C**) KEGG, GO and GSEA (**D**) analysis of DEGs.

Based on the given information, there were a total of 501 differentially expressed coding genes between the BRCA1 high expression and low expression groups. Out of these genes, 317 were upregulated, accounting for 63.3% of the total, while 184 were downregulated, representing 36.7% (with an adjusted p-value<0.05 and | Log_2_ FC |>1) (Figure 6A). The study then focused on the top 10 DEGs and their relationship with BRCA1. These genes, including FGF4, CPLX2, PRSS48, SEZ6, IFNK, PAGE1, H4C13, DDI1, NEUROD4, and CSMD3, were further analyzed, and their association with BRCA1 was illustrated in Figure 6B. GO and KEGG enrichment analysis were conducted about all DEGs. The results revealed that BP (Biological Process) was mainly enriched involved in detection of chemical stimulus involved in sensory perception, detection of chemical stimulus involved in sensory perception of bitter taste and sensory perception of bitter taste. Regarding the cellular component (CC), nucleosome, CENP-A containing nucleosome and CENP-A containing chromatin were the major enrichments. For molecular function (MF), the enriched terms were bitter taste receptor activity, taste receptor activity and olfactory receptor activity. In terms of KEGG pathways analysis, the most significantly enriched pathways were Olfactory transduction, Neuroactive ligand-receptor interaction and Systemic lupus erythematosus (Figure 6C and Supplementary Table 1). Subsequently, GSEA demonstrated that DNA Double Strand Break Response biological processes were vitally enriched in the high BRCA1 level group (Figure 6D).

### The relationship between BRCA1 expression and methylation

**Figure 7:**
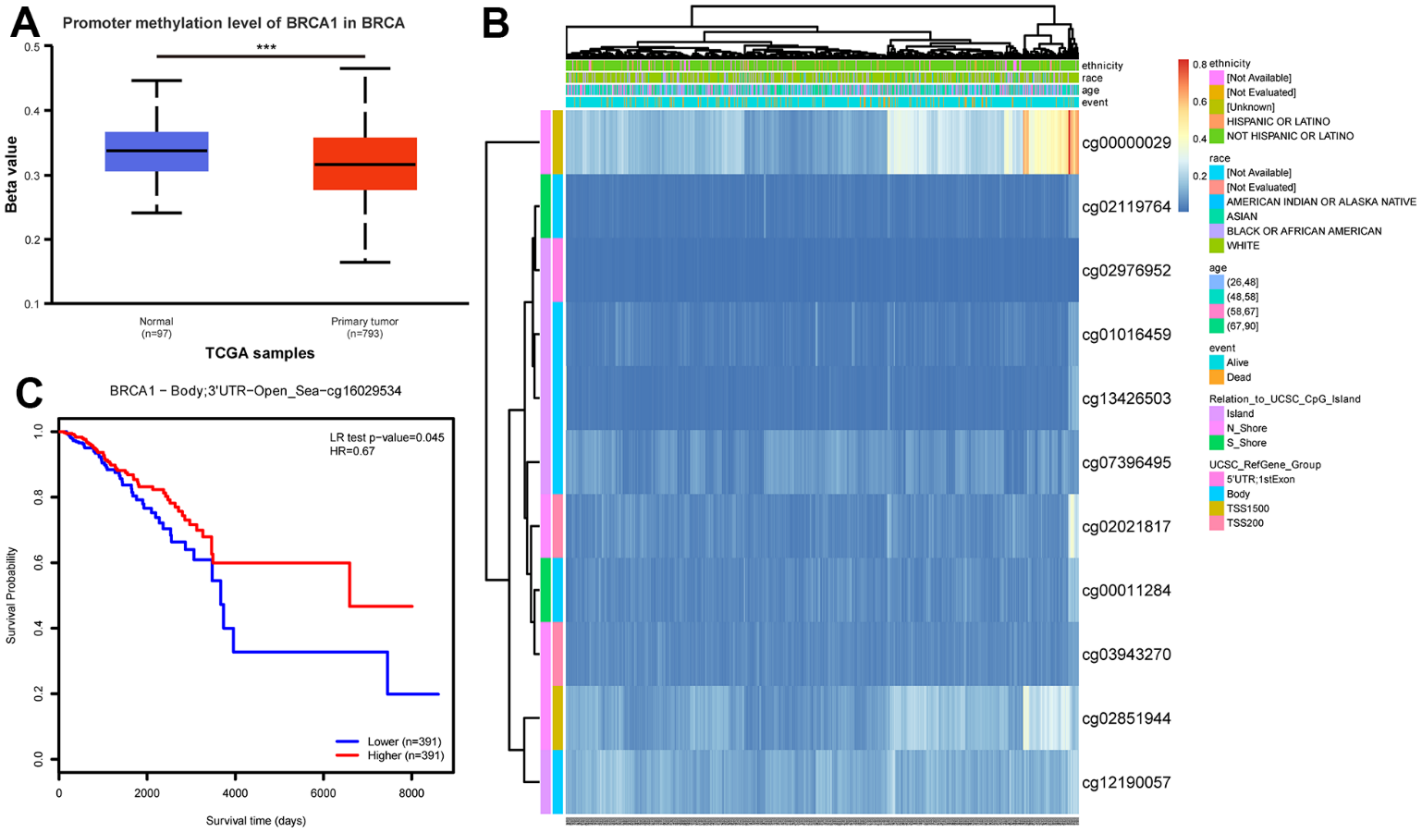
**DNA promoter methylation level of BRCA1 and its effect on prognosis of patients with BRCA.** (**A**) The promoter methylation level of BRCA1 in BRCA was lower than that in normal breast tissue. (**B**) Correlation between BRCA1 mRNA expression level and methylation level. (**C**) KM survival curves for methylation sites of BRCA1. P-values were calculated with two-tailed unpaired Student’s t-test, *p < 0.05, **p < 0.01, ***p < 0.001.

In order to clarify the potential mechanism of BRCA1 overexpression in BRCA, we also used online tools to study the correlation between BRCA1 expression level and methylation status. First, using the UALCAN database, we observed that the level of DNA methylation at the promoter in BRCA tissue was significantly lower than that in normal breast tissue (p<0.001) (Figure 7A). Most of the methylation sites in the BRCA1 DNA sequence were hypomethylated in BRCA. BRCA patients with low BRCA1 methylation had lower OS rates than those with high BRCA1 methylation (Figure 7B, 7C).

### Relation between BRCA1 and immune infiltration

**Figure 8:**
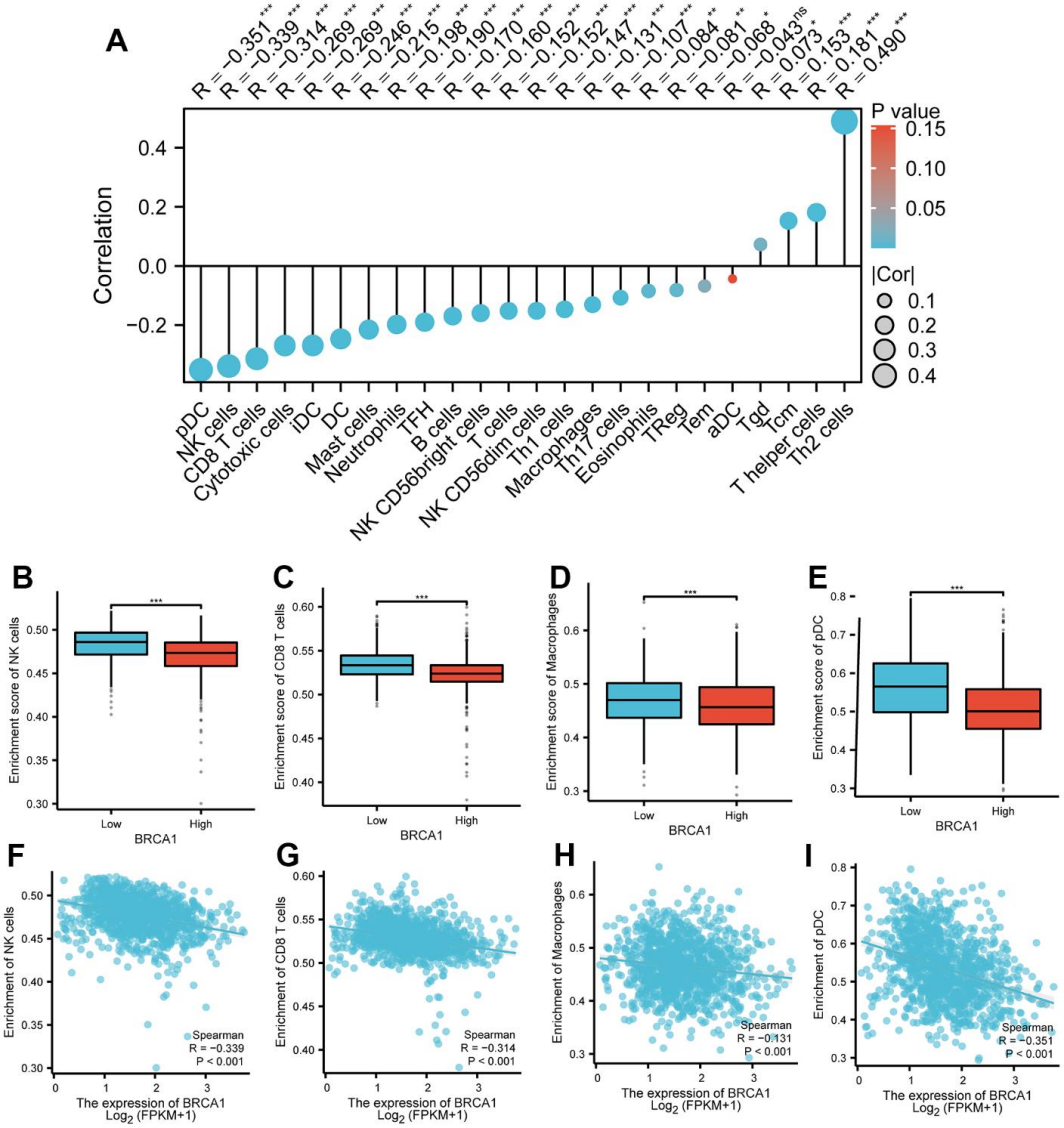
**Correlation between BRCA1 level and immune cells infiltration in BRCA.** (**A**) Correlation between BRCA1 expression and 24 types of immune cells. (**B**–**E**) Comparison of immune infiltration levels of immune cells (including macrophages, NK cells, pDC, and CD8^+^ T cells) between high and low BRCA1 level groups. (**F**–**I**) The expression of BRCA1 was negatively correlated with the level of infiltrating immune cells, including CD8^+^ T cells, NK cells, macrophages, and pDC. P-values were calculated with two-tailed unpaired Student’s t-test, *p < 0.05, **p < 0.01, ***p < 0.001.

The results displayed an apparent negative association between the level of BRCA1 and the number of immune cells infiltration, specifically macrophages, NK cells, pDCs and CD8^+^ T cells, and as shown in Figure 8A. Furthermore, the BRCA1 high level group exhibited substantially lower enrichment scores for CD8^+^ T cells, macrophages, NK cells, and pDCs compared to the BRCA1 low level group as illustrated in Figure 8B–8I. Additional, we were surprised to find a positive correlation between BRCA1 and PD-L1 (CD274) expression in BRCA (P<0.05) (Supplementary Figure 1).

### Construction and validation of a nomogram based on the independent factors

**Figure 9:**
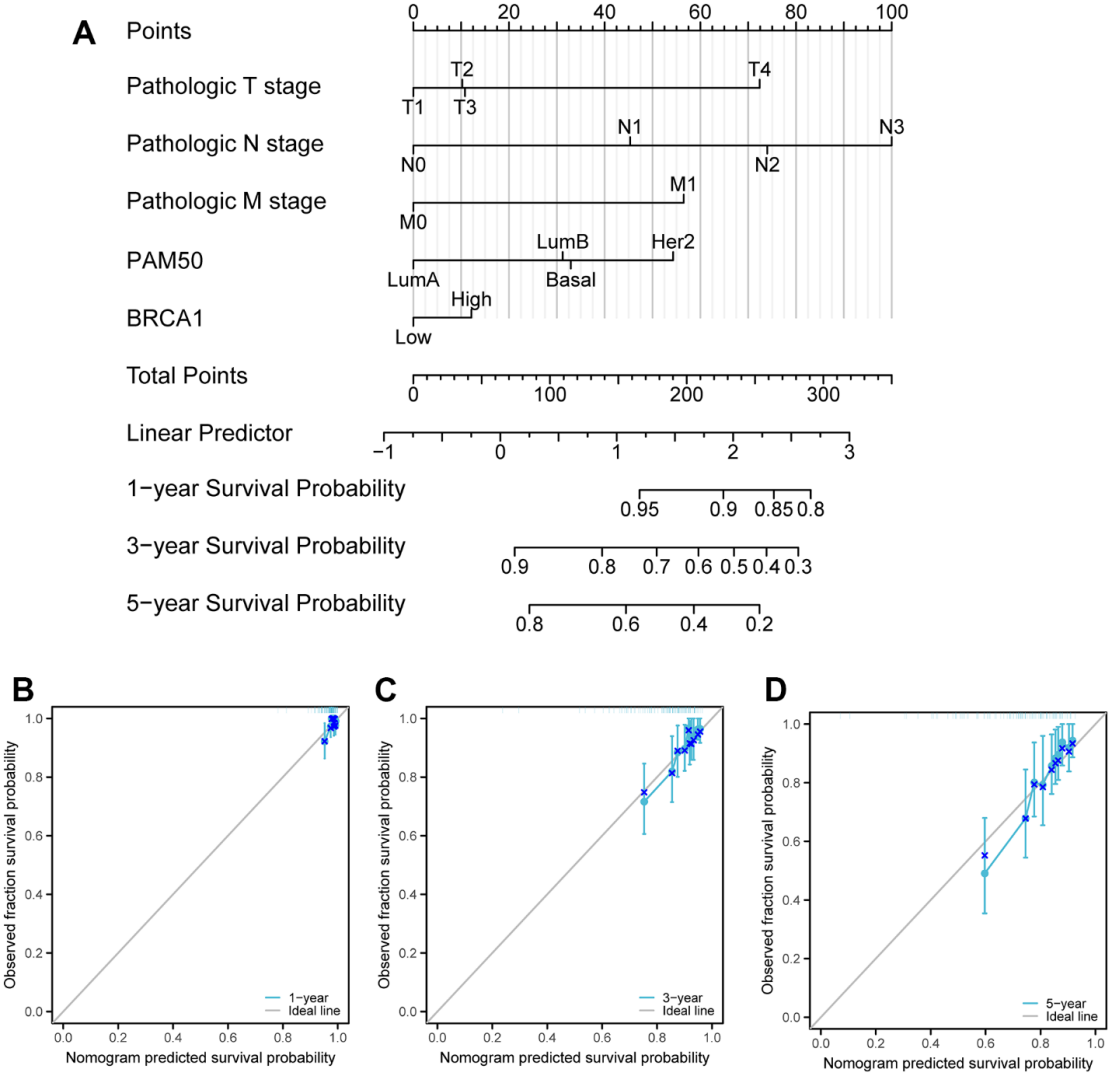
**Calibration curves and a nomogram and for prediction OS rates of BRCA patients.** (**A**) A nomogram chart was a visual representation that displays the data of BRCA patients’ OS rates at specific time intervals, such as one, three, and five years. (**B**–**D**) Calibration curves were graphical tools used to predict the survival rates of cancer patients at specific time points.

To predict the prognosis of BRCA patients, a detailed analysis was conducted using various independent factors to generate a comprehensive nomogram. It was observed that as the total number of points on the chart increased, indicating the presence of more adverse factors, the prognosis of the BRCA patients worsened. This was evident in Figure 9A, where higher total points were associated with poorer prognosis. Furthermore, calibration curves were employed to assess the accuracy and reliability of the predictive performance of the nomogram (Figure 9B–9D). Based on the results obtained from the analysis, it was found that BRCA1, a gene associated with BRCA susceptibility, played a crucial role as an independent prognostic factor in affecting the prognosis of patients with BRCA.

## DISCUSSION

Because of the heterogeneous nature of BRCA, the current predicted pathological indicators like grade, HER2, ER, Ki67 and PR have some limitations in predicting prognosis. It is urgent to discover new biomarkers that can improve prognosis prediction with BRCA and personalized treatment. In this study, we analyzed the expression of BRCA1 in BRCA using data from the TCGA database. We found that BRCA1 was highly expressed in various tumor types, including BRCA (Figure 1A–1C). In addition, the significant discrepancy of BRCA1 expression between BRCA and normal breast cells or tissues was proved by our experiment (Figure 2). Furthermore, the Receiver Operating Characteristic (ROC) curve analysis indicated that the expression of BRCA1 gene in BRCA patients demonstrated a high level of accuracy in predicting the presence of the disease. The Area Under the Curve (AUC) value of the ROC curve, which measures the discriminatory power of the BRCA1 expression in distinguishing between BRCA patients and healthy individuals, was determined to be 0.766 (Figure 1D). Similarly, BRCA1 had been considered a candidate oncogene and has been found to be highly expressed in several other types of tumors, such as digestive system cancers [16], ovarian cancer [17, 18], lung adenocarcinoma [19]. Therefore, we believed that the expression of BRCA1 was one of the new biomarkers for the diagnosis of BRCA.

In this study, our results displayed that the high level of BRCA1 in BRCA was related to some unfavorable clinicopathologic indicators, such as T2, IDC, ER positive, Stage II and PR positive (Figure 3A–3G). Moreover, the elevated level of BRCA1 in BRCA was an independent prognostic factor of poor OS. Similarly, further studies have discovered that BRCA1 level was a prognostic biomarker of poor survival in some solid tumors patients, including NSCLC [20, 21], ovarian cancer [22, 23], hepatocellular carcinoma [24], uterine serous carcinoma [25], and nasopharyngeal carcinoma [26, 27]. One potential explanation for the observed association between high BRCA1 expression and a poor prognosis in patients with BRCA is that elevated levels of BRCA1 could play a protective role in preventing additional DNA damage in cancer cells [28, 29]. This increased protection against DNA damage might then contribute to the development of resistance to chemotherapy treatments [30, 31], making it more difficult to effectively target and eliminate the tumor cells.

Many scientific studies have provided evidence demonstrating the impact of tumor microenvironment on the progression of tumors [32]. It has been found that the interaction between tumor cells and the microenvironment can greatly influence tumor growth, invasion, and the response to therapy [33]. DNA methylation, which contributes to decrease level of gene, is closely related to tumor microenvironment [34, 35]. In our work, we researched the potential mechanism of BRCA1 high level in BRCA, and discovered that the increase of BRCA1 may be correlated with DNA hypomethylation of BRCA1 (Figure 7A). Compared with hypermethylation BRCA patients, low methylation BRCA patients with BRCA1 had a poorer prognosis (Figure 7C). Additionally, immune cells, such as CD8^+^ tumor infiltrating lymphocyte (TIL), NK cells, pDCs, and macrophages, act a significant role in the body’s immune response against tumors in tumor microenvironment [36–39]. Research has consistently shown that the presence of these immune cells is indicative of an anti-tumor immune response. In our study, we found that the expression of BRCA1, a gene associated with BRCA, was negatively related to the number of infiltrating macrophages, CD8^+^ T cells, NK cells, and pDCs (Figure 8). The type, density, and location of immune cells within the tumor microenvironment could influence patient outcomes [39]. Furthermore, infiltrating immune cells have been identified as independent prognostic factors in response to PD-1/PD-L1 blockers therapies (immune checkpoint inhibition therapies) and neoadjuvant chemotherapy [40]. Specifically, the increase of infiltrating CD8^+^ T cells could improve the prognosis of BRCA patients [41]. These findings are consistent with the results of our research, suggesting that the high level of BRCA1 may influence prognosis of BRCA through modulating the number of infiltrating immune cells. Furthermore, we found a positive correlation between BRCA1 and PD-L1 expression in BRCA (Supplementary Figure 1). Therefore, we speculated that BRCA1 affected the infiltration of immune cells by increasing PD-L1 level.

Although this research provides a new view into the correlation between the level of BRCA1 and the prognostic price of the patients with BRCA, there are nonetheless a lot of shortcomings to be considered. Firstly, our research only concerned one dataset, which can also have restricted the variety of the patient populace and led to selection bias. Secondly, most of the data was downloaded from online databases. We were unable to assess the chemotherapy regimen received by the patients, which could potentially impact their prognosis and BRCA1 expression. In subsequent studies, a large number of experiments are needed to verify the results of this study.

In a word, this study has offered evidence suggesting that the presence of BRCA1 is associated with a worse prognosis in patients with BRCA. We found a strong correlation between BRCA1 and some invasive clinical features, such as tumor size, lymph node involvement. Additionally, we observed a negative impact of BRCA1 on immune cells infiltration, indicating that the presence of this gene may impair the body’s ability to mount an effective immune response against the tumor. Based on these findings, the research team proposed that BRCA1 could potentially serve as a new prognostic biomarker for BRCA. However, it is important to note that the exact mechanism by which BRCA1 influences the initiation and progression of cancer tumors remains unclear. Further investigation is required to unravel the complex biological pathways through which BRCA1 exerts its effects.

## Supplementary Material

Supplementary Figure 1

Supplementary Table 1
