# Supplementary material for: Establishing the role of BRCA1 in the diagnosis, prognosis and immune infiltrates of breast invasive cancer by bioinformatics analysis and experimental validation
Source: Aging (Albany NY). 2024 Jan 13;16(2):1077–95. doi: 10.18632/aging.205366 (PMC10866431; doi:10.18632/aging.205366)
Supplement: Supplementary Figure 1 [file aging-16-205366-s001.pdf]

SUPPLEMENTARY FIGURE

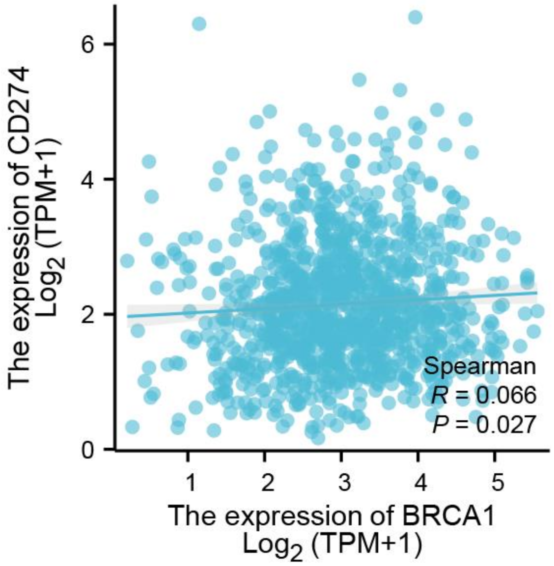

Supplementary Figure 1. Correlation analysis between the level of BRCA1 and CD274 using bioinformatics techniques. BRCA1 and CD274 are positively correlated in BRCA.
