# Supplementary material for: Establishing the role of BRCA1 in the diagnosis, prognosis and immune infiltrates of breast invasive cancer by bioinformatics analysis and experimental validation
Source: Aging (Albany NY). 2024 Jan 13;16(2):1077–95. doi: 10.18632/aging.205366 (PMC10866431; doi:10.18632/aging.205366)
Supplement: Supplementary Table 1 [file aging-16-205366-s002.pdf]

SUPPLEMENTARY TABLE

Supplementary Table 1. Functional enrichment analyses.

ONTOLOGY	ID	Description
BP	GO:0050907	detection of chemical stimulus involved in sensory perception
BP	GO:0001580	detection of chemical stimulus involved in sensory perception of bitter taste
BP	GO:0050913	sensory perception of bitter taste
CC	GO:0000786	nucleosome
CC	GO:0043505	CENP-A containing nucleosome
CC	GO:0061638	CENP-A containing chromatin
MF	GO:0033038	bitter taste receptor activity
MF	GO:0008527	taste receptor activity
MF	GO:0004984	olfactory receptor activity
KEGG	hsa04080	Neuroactive ligand-receptor interaction
KEGG	hsa04740	Olfactory transduction
KEGG	hsa05322	Systemic lupus erythematosus
